# Carriers of mitochondrial DNA macrohaplogroup R colonized Eurasia and Australasia from a southeast Asia core area

**DOI:** 10.1186/s12862-017-0964-5

**Published:** 2017-05-23

**Authors:** Jose M Larruga, Patricia Marrero, Khaled K Abu-Amero, Maria V Golubenko, Vicente M Cabrera

**Affiliations:** 1 Departamento de Genética, Facultad de Biología, Universidad de La Laguna, E-38271 La Laguna, Tenerife, Spain; 2 Research Support General Service, Universidad de La Laguna, E-38271 La Laguna, Tenerife, Spain; 3 Glaucoma Research Chair, Department of Ophthalmology, College of Medicine, King Saud University, Riyadh, Saudi Arabia; 4 The Research Institute for Medical Genetics, 634050 Tomsk, Russia

**Keywords:** Human evolution, Mitochondrial DNA, Macrohaplogroup R, Haplogroup U, Out-of-Africa

## Abstract

**Background:**

The colonization of Eurasia and Australasia by African modern humans has been explained, nearly unanimously, as the result of a quick southern coastal dispersal route through the Arabian Peninsula, the Indian subcontinent, and the Indochinese Peninsula, to reach Australia around 50 kya. The phylogeny and phylogeography of the major mitochondrial DNA Eurasian haplogroups M and N have played the main role in giving molecular genetics support to that scenario. However, using the same molecular tools, a northern route across central Asia has been invoked as an alternative that is more conciliatory with the fossil record of East Asia. Here, we assess as the Eurasian macrohaplogroup R fits in the northern path.

**Results:**

Haplogroup U, with a founder age around 50 kya, is one of the oldest clades of macrohaplogroup R in western Asia. The main branches of U expanded in successive waves across West, Central and South Asia before the Last Glacial Maximum. All these dispersions had rather overlapping ranges. Some of them, as those of U6 and U3, reached North Africa. At the other end of Asia, in Wallacea, another branch of macrohaplogroup R, haplogroup P, also independently expanded in the area around 52 kya, in this case as isolated bursts geographically well structured, with autochthonous branches in Australia, New Guinea, and the Philippines.

**Conclusions:**

Coeval independently dispersals around 50 kya of the West Asia haplogroup U and the Wallacea haplogroup P, points to a halfway core area in southeast Asia as the most probable centre of expansion of macrohaplogroup R, what fits in the phylogeographic pattern of its ancestor, macrohaplogroup N, for which a northern route and a southeast Asian origin has been already proposed.

**Electronic supplementary material:**

The online version of this article (doi:10.1186/s12862-017-0964-5) contains supplementary material, which is available to authorized users.

## Background

Although mitochondrial DNA (mtDNA) is only a small molecule with maternal inheritance, it has played a main role in the interpretation of the human evolution. The recent origin of early modern humans in Africa and their subsequent spread across Asia and Australasia, replacing other hominins when dwelling there, was first outlined comparing African and Eurasian levels of mtDNA polymorphism [1, 2]. After an initial strong opposition from the multiregional field [3], the hypothesis of a single and recent African origin of modern humans has finally gained a multidisciplinary agreement [4]. The recent evidence provided by ancient DNA studies of minor hybridization of modern humans with other hominins as Neanderthals [5, 6] and Denisovans [7, 8], has been considered as the result of a very low rate of interbreeding (2–5%) compatible with the replacement scenario. Nonetheless, the apparent continuous evolution of human fossils and their cultural remnants in East Asia during the whole Pleistocene is still interpreted as proof that this area was one of the origins of modern humans [9–11]. The time and migration routes took by modern humans out of Africa have also been proposed from the phylogeny and phylogeography of mtDNA lineages across Eurasia and Australasia. Although the fossil record pointed to the Levant as the most obvious path, the delayed colonization of Europe compared to Australia and the detection in East Africa of mtDNA M lineages that were absent in western Eurasia but predominant in India and eastern Asia [12], gave rise to the coastal southern route hypothesis, suggesting a single dispersal wave out of Africa of modern humans crossing the Bab el-Mandab strait from the Horn of Africa to southern Arabia and then, coasting the Indian Ocean, quickly reached southeast Asia and Australia. In this context, the colonization of Europe was considered a late offshoot of this route [13]. Surprisingly, in spite of any fossil evidence corroborating this idea and against the regressive trend in lithic technology evidenced by the archaeological record [14] this hypothesis has been enthusiastically followed by the bulk of geneticists to explain their interesting sets of genetic data gathered from Asian and Austronesian indigenous populations [15–19]. Against the mainstream, an alternative northern route across the Levant that carried the mtDNA macrohaplogroup N to Australia has been proposed long ago [20, 21]. This idea has been recently revived by the support given by new genetic data and data gathered from other disciplines [22]. Furthermore, based on mtDNA phylogenetic and phylogeographic grounds, it has been also suggested that mtDNA macrohaplogroup M most probably entered India from eastern Asia, in opposition to the eastward migration proposed by the southern route supporters [23]. These previous articles have satisfactorily explained the lack of autochthonous macrohaplogroup N (xR) lineages in India and the lack of primitive macrohaplogroup M lineages at the northwestern side of the Himalayas. The absence of recombination makes mtDNA especially amenable to genealogical treatment. The coalescence of all extant mtDNA macrohaplogroup L lineages gave a date of around 150–250 thousand years ago (kya) for the common ancestor of all humans in Africa. Applying the same approach to the extant M and N lineages, which comprise all the mtDNA diversity in the rest of the world, gave a coalescence date of around 50–65 kya that has been considered as the time frame for the out of Africa dispersal of modern humans [24, 25]. These phylogenetic inferences are at odds with dates obtained from the fossil record that point to the presence of modern humans around 100 kya in the Levant [26], and in southern China [11, 27–29]. The usual genetic explanation for this discrepancy is that these fossils have not left any genetic contribution to extant humans at least from a mtDNA maternal perspective. In turn, paleoanthropologists question the consistency and absolute value of the mutation rate. In the near future, ancient DNA will probably mediate on this issue. Meanwhile, the ancient DNA analysis of a 40,000-year-old Tianyuan fossil, anatomically classified as an early modern human, resulted in a genetically fully modern human carrying a haplogroup B mtDNA lineage already derived from the Eurasian macrohaplogroup N [30].

Unlike M and N, the third Eurasian mtDNA macrohaplogroup R presents indigenous worldwide distributions out of Africa. In this article, our main objective is to integrate the phylogeny and phylogeography of macrohaplogroup R, with their M and N Eurasian counterparts, into a congruent northern dispersal route of early modern humans out of Africa. To this end, we first compared the coeval expansion of two R haplogroups, haplogroup U in western Eurasia and haplogroup P in Near Oceania. For the case of haplogroup U, we analyzed 69 unpublished sequences of its U3 branch and completely sequenced 41 of them to improve its phylogeny. To put U3 into phylogenetic context we added 15 additional unpublished complete sequences belonging to the major branches of macrohaplogroup U. For haplogroup P we could only add one unpublished complete sequence belonging to the Philippine clade P9. Phylogeographic analysis for U3 was based on 1328 U3 partial sequences, given special attention to the later colonization of Africa by the carriers of this Eurasian haplogroup. For the phylogeographic analysis of the rest of macrohaplogroup R branches, we used 109,497 already published partial or complete sequences.

## Material and methods

### Sampling information

For the specific haplogroup U3 analysis a total of 103,313 partial sequences of worldwide origin were screened (Additional file 1: Table S1), of them 2757 belong to our unpublished data. We obtained a total of 1328 samples of U3 ascription of which 69 were from our unpublished records (Additional file 1: Table S1). For phylogeographic and population genetics analysis we worked with a total of 1017 U3 sequences, after excluding those belonging to Roma/Gypsies and Jews because of their uncertain geographic origin, and those of Indian origin since we considered this area as a secondary center of U3 expansion (Additional file 1: Table S2). To fully characterize these samples, 41 complete mtDNA U3 genomes were sequenced (Additional file 1: Table S3). We enlarged our U3 tree (Additional file 2: Figure S1) with 10 published complete sequences because they present a transversion (AY882383) or a reversion (AY882385, HQ404665, JX153143) at the diagnostic U3 transition at np 16,343, or belong to poorly characterized lineages as U3b2a1 (EU935438) and U3c (HM852803), or have mutations in the regulatory region that help to assort partial U3 sequences into their more probable clades as those with 16,104 (KC851932), 16,148 (KJ445940), 16166dA (JN969086), 16266G (AY714023) and 16,274 (AY882383). In addition, we added to the tree 15 unpublished complete mtDNA genomes, belonging to the main lineages of haplogroup U (Additional file 1: Table S3), to put the U3 sequences into phylogenetic context (Additional file 2: Figure S1). For the specific haplogroup P study, only one (Additional file 1: Table S3), unpublished complete sequence of the Philippine lineage P9a (Flp107) could be added to the haplogroup phylogeny (Additional file 2: Figure S2). However, a total of 10,962 complete or partial mtDNA published sequences were screened, 10,591 belonging to the West Pacific Islands (Additional file 1: Table S4) and 371 belonging to the Australian continent (Additional file 1: Table S5). For the global macrohaplogroup R study, we screened 109,497 already published partial and complete sequences (Additional file 1: Table S6) that largely overlap those used to the specific U3 study (Additional file 1: Table S1). All the above-described sequences were used to obtain the relative frequencies of macrohaplogroup M, N, and R in the main regions of Eurasia and Australasia (Additional file 1: Table S7). Human population sampling procedures followed the guidelines outlined by the Ethics Committee for Human Research at the University of La Laguna and by the Institutional Review Board at the King Saud University. All the samples were collected in the Canary Islands or in Saudi Arabia from academic and/or health-care centers. Written consent was recorded from all participants prior to taking part in the study.

### MtDNA sequencing

Total DNA was isolated from buccal or blood samples using the POREGENE DNA isolation kit from Gentra Systems (Minneapolis, USA). The mtDNA hypervariable regions I and II of the U3 samples were amplified and sequenced for both complementary strands as detailed elsewhere [31]. When necessary for unequivocal assortment into specific subclades, the U3 samples were additionally analyzed for haplogroup diagnostic SNPs using partial sequencing of the mtDNA fragments including those SNPs, or typed by Snapshot multiplex reactions [32]. For mtDNA genome sequencing, amplification primers and PCR conditions were as previously published [20]. Successfully amplified products were sequenced for both complementary strands using the DYEnamic™ETDye terminator kit (Amersham Biosciences) and samples run on MegaBACE™ 1000 (Amersham Biosciences) according to the manufacturer’s protocol. The 57 new complete mtDNA sequences have been deposited in GenBank under accession numbers KY411439-KY411495 (Additional file 1: Table S3; Additional file 2: Figures S1 and S2).

### MtDNA macrohaplogroup R sequences compilation

Sequences belonging to specific R haplogroups were obtained from public databases such as NCBI, MITOMAP, the 1000 Genomes Project and from the literature. We searched for mtDNA lineages directly using diagnostic SNPs (http://www.mitomap.org), or by submitting short fragments including those diagnostic SNPs to a BLAST search (http://blast.st-va.ncbi.nlm.nih.gov/Blast.cgi). Haplotypes extracted from the literature were transformed into sequences using the HaploSearch program [33]. Sequences were manually aligned and compared to the rCRS [34] with BioEdit Sequence Alignment program [35]. Haplogroup assignment was performed by hand, screening for diagnostic positions or motifs at both hypervariable and coding regions whenever possible. Sequence alignment and haplogroup assignment were carried out twice by two independent researchers and any discrepancy resolved according to the PhyloTree database Building 17 [36].

### Phylogenetic analysis

Phylogenetic trees were constructed by means of the Network v4.6.1.2 program using the Reduced Median and the Median Joining algorithms in sequent order [37]. Resting reticulations were manually resolved attending to the relative mutation rate of the positions involved [38]. Haplogroup branches were named following the nomenclature proposed by the PhyloTree database [36]. Coalescence ages were estimated by using statistics rho [39] and sigma [40], and the calibration rate proposed by [38]. Differences in coalescence ages were calculated by two-tailed t-tests. It was considered that the mean and standard error estimated from mean haplogroup ages calculated from different samples and methods were normally distributed.

### Phylogeographic analysis

In this study, we are dealing with the earliest periods of the out-of-Africa spread. Given that subsequent demographic events most probably eroded those early movements, we omitted spatial geographic distributions of haplogroups based on their contemporary frequencies or diversities. The presence/absence of R basal lineages, omitting regions with only derived branches or regions of known recent colonization, was used to establish the present-day geographic range of each haplogroup. We used the geometric center of these areas as the hypothetic center of dispersion for each haplogroup and defined it by its geographic coordinates. After that, to situate the hypothetic focus of the primitive radiation of R* in the whole area, we averaged the latitudinal and longitudinal coordinates of the evolved R lineages and take it as the center of the first dispersion of R* in the whole area.

### Geographic partition of Eurasia and Australasia

In order to assess geographic mtDNA haplogroup prevalence and relative overlapping we have considered the following geographic areas: West/Central Asia (WCA): taking all European countries, all western Asian countries and all central Asian countries including Afghanistan and Tibet. South Asia (SA): comprising Pakistan, India, Bhutan, Nepal, Bangladesh and Sri Lanka. East Asia (EA): Mongolia, China, Koreas, Japan and eastern and northern Siberia. South East Asia (SEA): All mainland and insular countries excluding the Indonesian Papua. Near Oceania (NO): The whole New Guinea Island including Indonesian Papua, and surrounding archipelagos. Australia (AU): considering only indigenous samples.

### Geographic subdivision of India and regional haplogroup assignation

India was subdivided into four different sampled areas: Northwest, including Kashmir, Himachal Pradesh, Punjab, Haryana, Uttarakhand, Rajasthan, Uttar Pradesh, Gujarat, and Madhya Pradesh states; Southwest, including Maharashtra, Karnataka and Kerala; Northeast including Bihar, Sikkim, Arunachal Pradesh, Assam, Nagaland, Meghalaya, Tripura, Jharkhand, West Bengal, Chhattisgarh and Orissa; and Southeast, represented by Andhra Pradesh, Tamil Nadu and Sri Lanka. We are very skeptical of the possibility that the actual genetic structure of India is the result of its original colonization, so the ethnic or linguistic affiliations of the samples were not considered but only its geographic origin. For the same motif we did not use the present day frequency and diversity of the haplogroups but their geographic ranges and radiation ages. The criteria followed to assign haplogroups to different regions within India were: consistent detection in an area (at least 90% of the cases) and absence or limited presence in the alternative areas (equal or less than 10% of the cases). We considered widespread those haplogroups consistently found in all the Indian areas and also found in surrounding areas as Pakistan or Iran to the west, Tibet or Nepal at the north, and Bangladesh or Myanmar at the east.

### Geographic assignation of mtDNA haplogroups

Following other authors, and after a confirmative clustering approach, we have assorted all the main mtDNA haplogroups into its most probable native areas as follows: a) West/Central Eurasia (WCA): N1, N2, N3, N5, X; R0, R1, R2, JT, U. b) South Asia (SA): M2, M3, M4, M5, M6, M18, M25, M30, M31, M32, M33, M34, M35, M36, M37, M38, M39, M42b, M61, M62, M63, M64, M65, M66, M67, M70, M81; R5, R6, R7, R8, R30, R31. c) East Asia (EA): M7, M8, M9, M10, M11, M12, G, D; N9, A; B, F, R11. d) Southeast Asia (SEA): M13, M17, M19, M20, M21, M22, M23, M24, M26, M46, M47, M50, M51, M55, M68, M69, M71, M72, M73, M74, M75, M76, M77, M78, M79, M80, M82, M83, M84, M90; N7, N8, N10, N11, N21, N22, N23; P9, P10, R9, R21, R22, R23, R24. e) Near Oceania (NO): M27, M28, M29, Q; P1, P2, P3b, P4a, R14. f) Australia (AU): M14, M15, M42a; N13, N14, O, S; P3a, P4b, P5, P6, P7, P8, R12.

### Population genetics analysis

Genetics distances, identities, and haplotype diversities, as well as AMOVA analysis, were calculated by means of the GenAlEx6.5 software [41]. For MANTEL tests and Pearson correlation analysis, we used the XLSTAT statistical software. PCA was performed using the Excel add-in Multibase package (Numerical Dynamics, Japan). Poisson distributions were implemented using the online calculator (http://stattrek.com). Maps and geographic coordinates were obtained by Google Earth software (https://earth.google.com).

## Results and discussion

### The spread of haplogroup U in west Eurasia with emphasis on branch U3

MtDNA haplogroup U has a prominent West/Central Eurasian geographic range. Its first radiation seems to have occurred in the initial stable warm phase of the MIS 3 interstadial around 50 kya (Table 1). We situated its hypothetical center of expansion in the Dahoguz province of Turkmenistan (Table 1). Next expansions, at around 43 and 33 kya, involved branches U1, U2 and U8 and branches U3, U5 and U6 respectively, and occurred also in periods of relatively mild temperatures. A more recent radiation was dominated by branches U4, U7, U9, and K around 24 kya, just before the Last Glacial Maximum. All these dispersion waves had rather overlapping ranges and preferably southward expansions [42]. Most probably, U1, U3, and U9 went to the Middle East [43–46]; U2i and U7 mainly to South Asia [47–49]; U2e, U4, U5 and U8 to Europe [50–52], and U6 to North Africa and the Mediterranean basin [53–57]. The recent analysis of the mitogenome of a 35 ky-old modern human from Romania, resulted in being a basic U6* sequence, supporting a Paleolithic Eurasian origin for this African lineage [58]. Secondary branches of these haplogroups have revealed more recent and limited human migrations in all the regions mentioned above. Interesting examples are the expansions to the Volga-Ural and Siberian regions of numerous sub-branches of U1a, U2e, U3b, U4, U5b, U7a, U8a and K1a [59, 60], to India of U2i and U7 [61], to Europe of K [62], or in North Africa of U5b [63]. Advances in ancient DNA technology have also made diachronic studies of human populations possible. An outstanding case was the genome sequencing of a 45 ky-old modern human from western Siberia that already carried a basic mtDNA U* lineage [64]. Haplogroups U4, U5, and U8 were the most prominent U lineages in Paleolithic hunter-gatherers of North and Central Europe, but its frequencies drastically diminished with the Neolithic expansion into the area [65–69], while other U lineages as U2, U3, and K, drastically increased in subsequent periods [66, 70–73]. The fact that Neolithic and Bronze Age migrations introduced southern Siberian U5a1b1e and U5b2a2 lineages to Ireland is noteworthy too [74]. It has been also reported the presence of western U2e, U5a and U7 lineages in prehistoric remains as far as the Tarim Basin and Northeast Mongolia [75, 76].

**Table 1:** Ages and geographic centre of West/Central Asia R haplogroups

Haplogroup	Mean age ky	Longitude E	Latitude N	Centre
R0	39.6 ± .6	40.198143	44.644106	Oktiabria
R1	58.6 ± .8	48.059934	46.358804	Astracan
R2	17.7 ± 2.2	66.974973	34.627012	Samarkanda
J	31.0 ± 4.4	47.149943	39.683415	Jarkov
T	24.3 ± 2.9	45.681486	43.316880	Grozni
U1	40.6 ± 7.7	44.380392	35.465576	Kirkuk
U5	28.7 ± 5.7	36.156224	51.709196	Kursk
U6	33.8 ± 4.9	-	-	-
U2	40.0 ± 12.4	74.872264	31.633979	Amritsar
U3	35.1 ± 3.7	49.589123	37.268218	Rasht
U3 (1)	34.7 (20.4/49.7)	-	-	-
U3a’c (1)	28.1 (15.3/41.7)	-	-	-
U3a (1)	13.4 (8.0/18.9)	-	-	-
U3c (1)	19.0 (13.5/24.7)	-	-	-
U3b (1)	20.6 (7.4/34.7)	-	-	-
U3b1 (1)	16.2 (4.1/29.1)	-	-	-
U3b2 (1)	22.8 (14.8/31.0)	-	-	-
U3b3 (1)	12.5 (2.0/26.0)	-	-	-
U4	22.6 ± 4.4	73.324236	54.988480	Omsk
U7	22.0 ± 7.3	71.633333	40.483333	Chimkent
U8 (xK)	49.3 ± 3.6	51.817392	44.243036	Shayyr
K	26.7 ± 3.8	59.616754	36.260462	Mashhad
U9	24.7 ± 1.1	69.207486	34.555349	Kabul
U	49.6 ± 1.6	58.955245 ± 14.928846	40.734181 ± 8.037318	Dahoguz
R	59.0 ± 0.2	55.675835 ± 13.243427	41.088417 ± 6.833329	Uzbekistan

(1) Based only in our data

Attending to our own data, it should be mentioned that our U9a Arab (Ara073) sequence is identical to a published (KM986517) Yemeni sample [77]. The Arab (Ara717), of U8b1a1 ascription, shares transversion 6515G and transition 10,632 with four (JX153780, JX153902, JX153925, KF161759) U8b Danish sequences [78, 79]. Arabs (Ara 224 and Ara 136), belonging to the U7a branch share, respectively, transitions 11,353, 14,110 and 15,218 with two Iranian [46] and one Pathan [80], and transition 16,234 with one Persian(9_IRE_PS) U7a lineage [46]. Curiously, our Arab (Ara815) U4d shares transitions 11,914 and 16,189 with a U4a sequence (JQ705609) and transition 6260 with a U4c sequence (JQ709993) pointing to parallel mutational events.

Focusing on haplogroup U3, this clade has transition 16,343 as a diagnostic position, however, it is rather unstable as evidenced by one transversion (AY882383) and three reversions (AY882385, HQ404665, JX153143) that occur in parallel in the phylogenetic tree (Additional file 2: Figure S1). Thus, searching U3 sequences using only this position might not be exhaustive enough. However, several HVSI positions are rather reliable to assign haplotypes to specific branches. Thus, transition 16,148 with 16,343 and 16,390 defines a new U3a northern African clade detected long ago in Mozabite Berbers [81]; transition 16,356, in the same 16,343, 16,390 background characterizes the U3a1c sub-branch. Also, the HVSII transition at np 200 is a good indicator for U3a2 ascription. The U3c branch has also a characteristic additional HVSI motif (16,193, 16,249, 16,526), although some of its haplotypes might lack the entire set. The presence of 16,086 is indicative of U3b1a membership, that of 16,168 of U3b3 and deletion 15944dT of U3b2. However, clades identified within U3b and in U3b2, based on the sharing of the 523dCA deletion, have to be considered as provisional due to the high instability of this mutation (Additional file 2: Figure S1).

Coalescence ages estimated for the main branches of U3 (Table 1) are, for the most part, comparable to those published previously [38, 46]. However, there are minor discrepancies. For instance, branch U3a’c is older in our study (28.1; 15.3/41.7 kya) compared to 18–26 kya in Derenko et al. [46], but in the last study U3c was represented by only one Azeri sequence that we have augmented by adding a Moroccan (Mor459) and a Jordanian (823) sample. On the contrary, sub-branch U3b3 (17.6; 8.8/26.8 kya) is older in Derenko et al. [46] than in this study (12.5; 5.0/26.0 kya) but, in this case, our U3b3 branch only comprises three Iberian and three North African peripheral sequences (Additional file 2: Figure S1) while those of Derenko et al. [46] belong to Iran and the Caucasus central areas. Finally, we have detected a new clade within the U3b1 branch defined by transition 2833 that includes three Iberian and one Jordanian sequence and that has a coalescence age of 14.8 (8.8; 20.9) ky. It seems that the majority of the U3 branches expanded in Paleolithic and Mesolithic periods astride the LGM. More recent dispersions occurred in Neolithic and subsequent periods as commented above.

Geographically (Table 2), basic (16343) U3*lineages are widespread but concentrate their highest frequencies in the Balkans, Eastern Europe, and Russia. U3a, mainly the U3a1 branch (defined by transition 3010), has a prominent European range, with special emphasis in northern, western and southern areas and a notable incidence in northwest Africa, which denotes a common colonization of both regions, perhaps, by maritime contacts since Neolithic times, as already suggested by the pattern of other mtDNA haplogroups and other genetic markers [31, 63, 82–90, 90]. At this respect, it is significant the presence of specific branches (U3a2) in the Middle East (Additional file 2: Figure S1). The U3c branch seems to be concentrated in the Caucasus, the Middle East and down to East Africa not involving, however, the Arabian Peninsula. On its hand, U3b is also most abundant in the Caucasus, Middle East and, in this case, the Arabian Peninsula and, after that, northwest Africa, pointing to a dual colonization of this region as previously envisaged [87, 88]. Haplotypic diversity is highest in the Middle East, the Arabian Peninsula and southern Europe, and haplotypic richness and percentage of exclusive haplotypes also peak in Arabia (Table 3), pointing to this Peninsula as an important center of expansion in the past. Correlations between U3 diversity and geographic coordinates showed that there is a significant negative correlation (*r* = −0.672; *p* < 0.012) only with latitude, clearly pointing to a more recent colonization of the northern areas. However, Mantel test results indicate that although pair-wise genetic distances and identities are negative and significantly correlated (*r* = −0.365; *p* < 0.0001), they are not so with its geographic distances, *p* = 0.298 and *p* = 0.071 respectively (Additional file 1: Table S8).

**Table 2:** Frequencies of U3 subhaplogroups in the different regions

Regions	Sample	U3*	U3a*	U3c*	U3b*
Arabia	34	0.088	0.294	0	0.618
East Africa	25	0.160	0.360	0.120	0.360
West Africa	62	0.032	0.500	0.016	0.452
Near East	148	0.338	0.311	0.040	0.311
Middle East	176	0.188	0.233	0.074	0.505
Caucasus	68	0.220	0.147	0.118	0.515
Central Asia	45	0.267	0.333	0	0.400
Russia	30	0.467	0.300	0	0.233
North Europe	51	0.176	0.667	0.020	0.137
West Europe	84	0.262	0.548	0.047	0.143
East Europe	29	0.448	0.173	0	0.379
The Balkans	79	0.430	0.342	0	0.228
South Europe	186	0.210	0.435	0.027	0.328

**Table 3:** Estimations of U3 genetic variability in the main regions studied

Region	Rho	Haplotypic diversity	Haplotypic richness (%)	Exclusive haplotypes (%)	Major haplotype*	Percent	Other abundant haplotypes	Percent
Arabia	2.53 ± 1.19	0.904	64.7	72.7	086119 (U3b1a)	17.6	343 (U3*)	8.8
East Africa	2.08 ± 1.15	0.881	64.0	43.8	343 (U3*)	16.0	148,343,390 (U3a)	12.0
West Africa	2.00 ± 1.04	0.890	30.6	52.6	260,343,390 (U3a)	17.7	148,343,390 (U3a)	16.1
Near East	1.37 ± 1.14	0.855	32.4	56.3	343 (U3*)	33.8	343,390 (U3a)	12.2
Middle East	1.57 ± 1.11	0.918	38.1	64.2	343 (U3*)	18.7	086343 U3b1a)	17.0
Caucasus	1.65 ± 1.00	0.804	33.8	30.4	168,192,343 (U3b3)	35.3	343 (U3*)	22.1
Central Asia	1.18 ± 1.39	0.841	31.1	35.7	343 (U3*)	26.7	343,390 (U3a)	20.0
Russia	0.90 ± 1.03	0.720	36.7	36.4	343 (U3*)	46.7	343,390 (U3a)	13.3
North Europe	1.74 ± 1.05	0.882	45.1	43.5	189 343,390 (U3a3)	19.6	343 (U3*)	17.6
West Europe	1.55 ± 1.11	0.868	38.1	40.6	343 (U3*)	26.2	343,390 (U3a)	17.9
East Europe	1.10 ± 1.15	0.746	48.3	28.6	343 (U3*)	44.8	343,390 (U3a)	6.9
The Balkans	0.94 ± 1.05	0.741	26.6	38.1	343 (U3*)	43.0	343,390 (U3a)	24.1
South Europe	1.62 ± 1.78	0.901	32.8	59.0	343 (U3*)	21.0	343,390 (U3a)	17.7

*minus 16,000

We think that results about haplogroup U point to a primitive maturation period of this clade in central/eastern regions of Eurasia. During this period it accumulated the three diagnostic mutations that differentiated it from other R lineages. After that, it branched out somewhere in Central Asia. Through long migrations, in unfavorable climatic conditions, and demographic expansions, in optimal environments, U has reached its present-day geographic range and phylogenetic ramification. No doubt about the important role played by the Arabian Peninsula as receptor of these successive migratory inputs and as centre of secondary expansions as we and others have evidenced by the analysis of other mtDNA haplogroups as R0a, before preHV1, [91–93]; J1 [45, 94]; N1a [77, 95, 96]; HV1 [97] and R2 [98]. However, we cannot find any mtDNA evidence in support of an Arabian Peninsula primary centre of expansion of the basic M, N, and R macrohaplogroups after the out-of-Africa exit of modern humans as proposed by others [96], neither for the Persian gulf, the new candidate suggested by the supporters of the southern route [99].

### The spread of haplogroup P in Wallacea

At the other end of Asia, east to the Wallacea line as proposed by Huxley on biological considerations, there is another R lineage, named P [100], native of this region. The coalescence age of P, around 52 kya, is at least as old as the one calculated for the western Asian haplogroup U (Additional file 1: Table S9). Haplogroup P is geographically well structured with branches P9 and P10 autochthonous to the Philippine Negrito populations [101–103]; branches P1 and P2 native of New Guinea aborigines [17, 104–106] and branches P5, P6, P7 and P8 exclusive of aboriginal Australians [17, 107–109, 109]. Finally, there are two additional clades, P3 and P4 that contain both New Guinean (P3b, P4a) and Australian (P3a, P4b) specific branches (Additional file 2: Figure S2). It has been stated that these lineages signal a deep common ancestry for the New Guinea and Australia first colonizers [17, 106]. The founder age of P in Near Oceania (55 kya) is slightly younger but not significantly different than the ones in the Philippines (62 kya) and Australia (61 kya). However, there are apparent differences (*p* = 0.001) in the average number of mutations accumulated on sequences of the Philippine P10 lineage (25.5) and those accumulated on the New Guinean P2 lineage (14.7) since both diverged from a common ancestral node, defined by a transition at np 3882 (Additional file 2: Figure S2). In the same way, there are significant differences (*p* = 0.033) in the mean branch length of the Australian P3a clade (8.5) compared to that of the New Guinean P3b sister branch (15.7), and also (*p* = 0.001) between the average number of mutations accumulated on the Australian P4b clade (15.3) and the ones (7.8) accumulated on its P4a sister branch in New Guinea (Additional file 2: Figure S2). Since differences in the substitution rate among different intraspecific clades were observed, alternative explanations were given favoring different selective pressures [110], or different population histories [53], acting on different lineages, although more complex scenarios have been invoked [111, 112]. In this case, as the regional age estimations of haplogroup P are rather similar, we think that population size differences in the colonization process, due to successive founder effects and successive expansions in relative isolation, is the best explanation for the heterogeneous mutation rate observed between sister clades in the different areas. Starting from a mother population fixed or nearly fixed for an R lineage with the haplogroup P diagnostic mutation at np 15,607 on the top of the common ancestral R root (12,705, @16,223), sequences will accumulate new mutations in a simple Poisson process, with a rate of success of one mutation every 3624 years for the entire molecule [38]. Even in a long interval of 10,000 years, the probability that no mutations occur in a sequence is 0.063 and that to accumulate five mutations is 0.084. Thus, in a population composed of one hundred females, even after 10,000 years of evolution, there could be around six females carrying the same mtDNA that their initial ancestors, and around eight females that have accumulated five new mutations. Now, if this population has gone through several bottlenecks due to successive subdivisions and expansions into new territories, it would be possible to find subpopulations separated by 10,000 km (supposing a rate of migration of 1 km per year for hunter-gatherers) that have lineages differing in five mutations in spite of its common origin. As the probability that the new founders carried some particular lineage is a function of its frequency in the mother population, we suppose that, on average, mutations on the migrants will accumulate later than on the source population. Under these premises, we might outline the colonization of Wallacea by carriers of haplogroup P. Most probably, macrohaplogroup R lineages differentiated from N in southeast Asia [22]. From them, the ones with haplogroup P* primitive status migrated to Wallacea, perhaps reaching the Philippines and the Australian part of the Sahul before than the highlands of New Guinea. This seems most probably because, whereas Guinean lineages accumulated an additional mutation (16176) before their spread, Australian migrants seem to have expanded since the very beginning as attested by the simultaneous sprout of independent lineages from the root (Additional file 2: Figure S2). Most likely, this number will grow when still unclassified Australian P* lineages be released [113]. Furthermore, the next older radiations of P also occurred in Australia, within P6 (52.8 ± 4.5 kya) and within P4 (58,2 ± 4.5) for which, as its oldest branch P4b is Australian, we propose an Australian origin too (Additional file 1: Table S9). The first expansion in the Philippines occurred later, within the P9 lineage around 46.5 kya, involving mainly ancestors of Aeta and Agta indigenous Negrito tribes from Luzon [102, 103], although members of this clade have been also detected in the Visayas and Manila [101]. Incidentally, our Flp107 P9 sample was voluntarily donated in the Gran Canaria harbor, by a Filipino fisherman from Cebu, 30 years ago. The place where the P3 radiation (37.2 ± 3.1 kya) originated is uncertain. Following our criterion of the most evolved branch, a New Guinean origin would be assigned. However, while the New Guinean sequences are specific of branch P3b, there are Australian sequences at both branches (Additional file 2: Figure S2). In addition, P3b is significantly (*p* = 0.0033) more abundant in the lowlands and islands of New Guinea than in the highlands where the highest diversity and frequency for the autochthonous lineages P1 and P2 occur (Additional file 1: Table S4). Thus, an Australian origin is also a possible alternative. More P3 sequences are needed to reach a more documented decision. Although younger, the star-like radiation of P2 in New Guinea (33,5 ± 1.2 kya) extended east and westwards to adjacent islands, with indigenous branches identified in Melanesia [106] and East Timor [114], and more erratic detections beyond both sides (Additional file 1: Table S4). The presence of isolated and derived P1d1 haplotypes in Australian Barrineans [115], in the Philippines [103], and in Malaysia [116], could reflect more recent, even historic contacts. Finally, we have the expansion of P2 (18.6 ± 3.9), a young New Guinean lineage that is a sister branch of the very much evolved P10 Philippine lineage (Additional file 2: Figure S2). Although the radiation of P10 is the youngest (5.2 kya), we think that a Philippine origin for the P2’10 ancestor is the most probable alternative. Within New Guinea (Additional file 1: Table S4), both P1 and P2 are significantly more abundant in the highlands than in the coast and nearby islands (*p* < 0.0001 in both cases), and less frequent in the West than in the East of the Island (*p* = 0.0026 for P1 and *p* = 0.0004 for P2). This peculiar distribution challenges both routes of expansion into Wallacea, that from the West through Timor as much as that from the North through the Moluccas. Turning to Australia, the still insufficient sampling of the Continent and the lack of full availability of some published data makes a phylogeographic approach difficult but, in spite of its incompleteness, the global distribution of haplogroup P in Australia (Additional file 1: Table S5) parallels that found in New Guinea, with significantly higher frequencies of P at the East compared to the West (*p* = 0.0002). Curiously, this is also the case for the Australian haplogroup M42 that shows significant higher frequencies (*p* < 0.0001) in the East of the Continent. This coincident distribution resembles that of P and Q in New Guinea [106], and the ancient implantation of autochthonous M haplogroups in northern Island Melanesia [117]. However, not all Australian mtDNA lineages fit this pattern. On the contrary, the less frequent haplogroup R12 is more abundant in the West (*p* = 0.0321) like other Australian M (XM42) rare lineages (*p* = 0.0087). Haplogroup R12 shares with R21 three mutations at sNPS 10,398, 11,404, and 16,295 as shown in Phylotree build17 [36]. As R21 was found first in Malaysian Negrito groups [15, 118, 119], later in Sumatra [120] and in Dayak from Borneo [121], its shared root would point to a possible common ancestor somewhere in Southeast Asia which is congruent with a western, a note of caution to this hypothesis is necessary because a Burman R* sequence (KP346030) found in Myanmar [19], shares with R21 also three mutations at NPS 1709, 1719, and 15,613. Counting homoplasies and reversions in phylotree build 17 for each trio of mutations, we obtained a total of 45 independent events for the R12/R21 root and a total of 44 for the R21/R* alternative. On the other side, another R branch (R14) went through the Lesser Sunda islands of Bali, Flores, Lembata, Timor [114, 118, 122, 123], and Borneo and Sulawesi [121, 123], reaching the highlands [17] and lowlands [124] of New Guinea following a pattern unlike to haplogroup P. There is still another branch (R24) indigenous of the Philippines [101, 103, 125, 126], also with a younger dispersion than P9 (Additional file 1: Table S9). Recently, this clade has also been detected in northern Moluccas [121]. Furthermore, the two main representatives of haplogroup N in Australia (O and S) have also significant higher frequencies (*p* < 0.0001 in both cases) in the West than the East of the Continent (Additional file 1: Table S5). This is in spite of the fact that S has been detected as far southeast as Tasmania [127]. Curiously, the two ancient indigenous Australians sequenced so far belonged to mtDNA haplogroup O [128] and S [129], having respectively a southwest and a southeast geographical origin. Autochthonous N lineages have not been detected in New Guinea or Melanesia. In the Philippines and Borneo the oldest N representatives are branches of the southern China lineages N11 and N10, respectively [18, 121, 126]. In Nusa Tenggara lineages N21 and N22 are younger than the N in Australia [22]. The Wallacea maternal pattern just described is congruent with the following scenario: 1) Like in other core areas of Eurasia, the first colonizers of this region carried basic M*, N* and R* mtDNA lineages that evolved independently and were unevenly affected by later Asian migrations; 2) Although supposing only one settlement wave would be the most parsimonious, there are hints that the colonization of Wallacea occurred at least in two waves, one from the North carried the M* and P* ancestors that settled the Philippines, New Guinea, Island Melanesia and eastern Australia; the other, from the West, reached northwestern Australia carrying distinct N* and R* maternal lineages. 3) Amazingly, we have to deduce that sea barriers were not an insurmountable obstacle for these primitive settlers. Naturally, a model is valid if it is not rejected by the results of other investigations. On the basis of Y-chromosome polymorphisms, a common origin and independent histories for Melanesia and Australia were proposed [130]. Also an unexpected Y-chromosome ancient diversity was uncovered in northern Island Melanesia [131], and in the Y-chromosome profile of Filipino, with some Negrito groups deeply associated to indigenous Australians [132]. Recent Y-chromosome genomic studies have confirmed the shared origin of aboriginal Australians and Papuans and also a deep divergence time between them of around 50 kya. It seems that only the Melanesian haplogroup M could be involved in more recent contacts with aboriginal Australians after 12 kya [133]. Also genome-wide analysis give congruent results, pointing to an early divergence of aboriginal Australians and Papuans from Eurasians around 51–75 kya [113, 128, 134], a deep common origin between them, and little evidence of substantial later migration, although a population expansion in northeast Australia past 10,000 years was inferred [113]. The singularity of Wallacea has also been inferred by the fact that the Denisova introgression in humans is 569 concentrated in this region, including the Philippines [135, 136, 136, 137].At the other hand, fossil hominid evidence from Wallacea points to the Philippines as the earliest point of entrance to the region at a minimum age of 67 ± 1 ky, if the hominin metatarsal recovered at the Callao cave in North Luzon is accepted as belonging to a modern human [138]. Finally, it should be emphasized that the earliest colonist of Australia owned a collection of simple industries including horse-hoof cores or tools that have been also detected in the Philippines, Borneo and Sulawesi [139]. More recent contacts between New Guinea and Australia could be envisaged by the sharing of edge-ground and waisted hatchets [140].The two examples of coeval mtDNA haplogroup R deep expansions in Western Asia (U) and Wallacea (P) are difficult to explain under the tenet of a single, rapid, coastal southern route out-of-Africa for the colonizers of Eurasia. Like for the cases of macrohaplogroups M [23] and N [22], supposing a northern route for R would better explain the phylogeny and phylogeography of this maternal lineage.

### Phylogeography and ages of macrohaplogroup R lineages

The main lineages of macrohaplogroup R are geographically well structured but, due to migration, secondary branches intertwined in transitional areas in such a way that population genetics approaches blur the phylogeny. Fortunately, coalescence going back in time returns clarity. Thus, the AMOVA analysis of 242 populations covering the main regions of Eurasia and Australasia (Additional file 1: Table S6) shows that 82% of the variance was found within populations and only 18% among regions (*p* < 0.0001). These results are graphically visualized in the PCA plot (Fig. 1) where the first (32.9%) and second (18.3%) components account for 51% of the variance. Clearly, Australia is the most isolated region, whereas Melanesia shows a small overlap with Island Southeast Asia, that, in turn, comprises some eccentric populations with anomalous haplogroup frequencies due to genetic drift. Haplogroups P and R12 have a major impact on this pattern. For Continental Eurasia a compact population continuous beginning in West Asia and finishing in Mainland Southeast Asia is observed, revealing important gene flow between adjacent regions. The western haplogroups R2’JT and R0 at the right and the eastern R11’B and R9’F at the left are the main responsible of this longitudinal population gradient. Only South Asia stands out with most populations composed of maternal indigenous haplogroups (R5–8/30,31).

Attending to the age of R and its main branches (Additional file 1: Table S9), there are not significant differences among the founder ages of macrohaplogroup R in the five major regions (Additional file 1: Table S9). However, mean radiation age in southeast Asia (17.6 ± 10.4) is significantly younger than those in West-Central Asia (51.2 ± 6.8), East Asia (48.8 ± 1.5) and Near Oceania (61.3 ± 6.4), but not different from the South Asia mean (47.0 ± 10.7) that, in turn, does not have differences with the rest of the regions. As in the case of macrohaplogroup N [22], the comparatively late expansion of R in southeast Asia is against the southern coastal route hypothesis and contrasts with the deep age of macrohaplogroup M in the area and with its westward gradual decline from Near Oceania to South Asia [23]. We traced the basic geographic ranges for the main Western/Central Eurasia R haplogroups, then, we obtained decimal coordinates for their geometric centers (Table 1). Averaging them, we calculated a hypothetical mid-point that we have considered the center of radiation for R in West-Central Eurasia. Coordinates for this center signals a core area between the Caspian Sea and the Pamir plateau (Table 1). On the other hand, all the authors, without exception, have situated the core area of expansion of the East Asian basic branches of R in southern China/northern Indochina [18, 21, 118, 141–145]. As we have discussed above, there was also a core area of R expansion in Wallacea. We still have the case of R in South Asia. Attending to their age we can consider two groups, one comprised by those with ages under 40 ky (R0, R5, R7 and R8) and the other with ages above 40 ky (R6, R30, R31, U, JT) with highly significant differences (*p* < 0.0001) between the mean age of each group, 36.8 ± 1.59 and 53.4 ± 2.69 ky respectively. On the other hand, attending to their geographic distribution, we have found a set of haplogroups (R0, R5, R6, R30, R31, J, T, U1, U5, U2”9) characterized because they are most frequent in northwest India and then in southeast India (*p* = 0.0154). On the contrary, haplogroups R7 [146, 147] and R8 [148] have their highest frequencies and diversities in northeast India. A third group (B, R1, R2, R21, R22, R9’F) has a prominent northern Indian range (*p* < 0.0001) most probably due to a later penetration in the Indian subcontinent. There is also the anecdotal case of the Pacific lineage P that is only detected in the southeast of India (Additional file 1: Table S6). Thus, we propose that R was introduced in India across the northwestern and the northeastern corridors. The northwestern colonizers, that came from the Pamir/Caspian core area, also expanded to West Asia reaching Europe later than India. Those from the northeast could arrive in India along with the carriers of haplogroup M [23]. However, all the N(xR) branches in India seems to have a northwestern origin [22]. Although numerous data favoring this dual colonization of South Asia have been reported previously [22, 23], we would like to add some more examples. EDAR (ectodysplasin-A-receptor) gene is a major genetic determinant of hair thickness [149]. Its 1540C allele shows high frequency in populations of East Asia but is essentially absent from Europeans and has low frequency in Melanesians [150]. However, in India, it has been observed in high frequencies, associated with Austro-Asiatic and Tibeto-Burman populations although it is absent among Indo-Europeans and Dravidians [151].Another case is the OAS 1 (2’-5’-Oligoadeylate Synthetase 1) gene that has a characteristic deep and diverse haplotype in Papuans that was, most probably, the result of introgression from Denisovan and seems restricted to eastern Indonesian and Melanesian populations. However, it has been occasionally detected in Pakistan and Sri Lanka. Analys is of comparative diversity suggested that this deep lineage migrated to South Asia from Melanesia [152]. On the other hand, it seems that the light skin phenotype at high latitudes evolved independently in East and West Eurasia [153]. However, it has been demonstrated that a lighter skin color in India is correlated with the frequency of the derived allele rs1426654-A of the SLC24A5 gene, that is nearly fixed in Europeans, whereas the African ancestral allele G predominates in East Asia. So, the light skin allele in South Asians and Europeans shares identity by descent [154]. Furthermore, it has been found that the rs142665-A allele has significantly higher frequencies in northern and northwest regions compared with Northeast regions of India, having the highest frequencies in Indo-Europeans populations and very low or virtually absent in Tibeto-Burman and Austro-Asiatic populations [154]. Finally, genome-wide analysis of the Indo-European Kalash, from northwest Pakistan, considered them as an extremely drifted ancient northern Eurasian population that also contributed to the European and Near Eastern ancestry [155].A puzzling question has been where L3 lineages evolved into M, N and R haplogroups in Eurasia. Our previous analysis strongly pointed to southeastern Asia, including southern China, as the most probable area [22, 23]. Congruent with this hypothesis, is the fact that the southern China core area is a geographic mid-point between the Central Asian and the Wallacea core areas described above for R. This is the region where the oldest N10 and N11 haplogroups first expanded. There is also the fact that the founder and expansion ages of R and N in the main regions are significantly correlated (*R* = 0.892; *p* = 0.0028). However, the lack of correlation between R and M (*R* = 0.354; *p* = 0.315) points to an independent, although overlapping expansion for macrohaplogroup M, perhaps, from an, even more, eastern geographic focus [23]. It has also to be stressed that mtDNA traces of Paleolithic East-to-West human expansion waves have been detected in Eurasia, highlighting the predominant role of eastern regions within Eurasia during Paleolithic times [156]. Moreover, the definitive resolution brought by genomic sequencing to the phylogeny and phylogeography of the Y-chromosome has also unveiled southeastern Asia as a primitive center of modern human expansion. A rapid diversification process of Y-chromosome haplogroup K-M526 has been detected in this area with subsequent westward expansions of the ancestors of haplogroups Q and R that originated the majority of paternal lineages of Central Asia and Europe [157]. Furthermore, it has been detected recently a rare F* lineage in Vietnam that is an out-group of a Y mega-haplogroup GHIJK-M3658 that comprises the Indian H. However, previous Indian F* lineages have been included in a clade designated H0 that split from the rest of haplogroup H [158–160]. A delayed expansion hypothesis has been formulated to explain the early divergence of Eurasian populations from Africans (90–110 kya) and the comparatively late divergence among different Eurasian populations (50–70 kya). According to this theory, an ancestral Eurasian founding population remained isolated long after the out-of-Africa diaspora before expanding throughout Eurasia [161]. A long journey of L3* maternal lineages since the out-of-Africa throughout Eurasia, its diversification into M, N and R lineages in southeastern Asia, and subsequent diasporas to repopulate Eurasia, as proposed previously [22, 23], and in this paper, for mtDNA fits extraordinarily well with the delayed scenario based on genomic diversity. Notice that this molecular landscape would help to incorporate into a coherent model the existence of modern humans in southern China, at least since 70 kya [11]. Furthermore, this mtDNA scenario also recovers contact with the brilliant hypothesis of Turner, who, based on dental variation, proposed southeastern Asia as the center of the evolution of modern humans [162].Finally, it should be mentioned that during the publication process, two new articles dealing with aspects mentioned here, have been published. In the first one [163] the authors suggest that haplogroup U7 had a very recent coalescence age, around 16–19 kya. However, its rho value based on complete mitogenomes (18.6 kya; 95% CI, 13.6–23.7 kya) widely overlaps with our own estimation (22.0 ± 7.3 kya). In addition, authors propose the Near East as the most probable expansion center of U7 while we situated the born of this haplogroup around southern Kazakhstan (Table 1). In the second article [164], authors intend to reconstruct the Australian mtDNA phylogeographic history before the European settlement. The strongly differentiated patterns detected between the East and West continental regions are highly coincident with those proposed in this study.

## Additional files

Additional file 1: Table S1. Worldwide mtDNA haplogroup U3 sequences. **Table S2.** MtDNA haplogroup U3 haplotypic frequencies (%) in Eurasian and northern Africa main regions. **Table S3.** MtDNA complete U and P sequences obtained in this study. **Table S4.** Mitochondrial DNA haplogroup P frequencies (%) in the West Pacific Islands. **Table S5.** Mitochondrial DNA haplogroup frequencies (%) in Australia. **Table S6.** Frequency (%) of major mtDNA macrohaplogroup R branches in different regions of Eurasia and Australasia. **Table S7.** MtDNA macrohaplogroup M, N and R frequencies (%) in Eurasia and Australasia. **Table S8.** Mantel tests based on correlations between geographic distances (a), genetic distances (b), and genetic identities (c). **Table S9.** Coalescence ages for the main branches of mtDNA haplogroup R in different geographic areas. (XLSX 266 kb)

Additional file 2: **Figure S1.** MtDNA haplogroup U phylogeny with emphasis on the U3 branch. **Figure S2.** MtDNA haplogroup P phylogeny. (XLSX 175 kb)
